# Antidepressant-Like Effects of Shuyusan in Rats Exposed to Chronic Stress: Effects on Hypothalamic-Pituitary-Adrenal Function

**DOI:** 10.1155/2012/940846

**Published:** 2012-09-13

**Authors:** Liping Chen, Mengli Chen, Fawei Wang, Zhigao Sun, Huang Quanzhi, Miao Geng, Hongyan Chen, Dongmei Duan

**Affiliations:** ^1^Department of Traditional Chinese Medicine, General Hospital of PLA, Beijing 100853, China; ^2^Department of Chinese Pharmaceutical, General Hospital of PLA, Beijing 100853, China; ^3^Postgraduate Medical School, General Hospital of PLA, Beijing 100853, China; ^4^Institute of Gerontology, General Hospital of PLA, Beijing 100853, China

## Abstract

This study was to investigate antidepressant activities of Shuyusan (a Chinese herb), using a rats model of depression induced by unpredictable chronic mild stress (UCMS). The administration groups were treated with Shuyusan decoction for 3 weeks and compared with fluoxetine treatment. In order to understand the potential antidepressant-like activities of Shuyusan, tail suspension test (TST) and forced swimming test (FST) were used as behavioral despair study. The level of corticotropin-releasing factor (CRH), adrenocorticotropic hormone (ACTH), corticosterone (CORT) and hippocampus glucocorticoid receptor expression were examined. After modeling, there was a significant prolongation of immobility time in administration groups with the TST and FST. High-dose Shuyusan could reduce the immobility time measured with the TST and FST. The immobility time in high-dose herbs group and fluoxetine group was increased significantly compared with the model group. After 3 weeks herbs fed, the serum contents level of CRH, ACTH, and CORT in high-dose herb group was significantly decreased compared to the model group. The result indicated that Shuyusan had antidepressant activity effects on UCMS model rats. The potential antidepressant effect may be related to decreasing glucocorticoid levels activity, regulating the function of HPA axis, and inhibiting glucocorticoid receptor expression in hippocampus.

## 1. Introduction

In studies of pathogenesis on depression, hypothalamic-pituitary-adrenal axis (HPA) hyperactivity, and neuroendocrine disorders are more recognized; in addition to monoamine neurotransmitters [1–3]. Hippocampus, as an important role of brain for cognition, emotion, and memory function, is the center of motional for learning and memory consolidation [4]. Studies have proposed that long-term chronic stress could cause hyperthyroidism of the HPA, that increased level of corticotropin-releasing factor (CRH), adrenocorticotropic hormone (ACTH), and corticosterone (CORT), which led to excessive expression of glucocorticoid receptor (GR) of HPA [5]. This long-term overexpression and increased levels of glucocorticoid caused by chronic stress could lead to hippocampus neuronal cell damage and lead to depressive symptoms, such as depressed mood, feeling of worthlessness, insomnia, forgetfulness, sexual dysfunction, and other symptoms of depression [6, 7].

Traditional Chinese medicine has a good effect in treatment of depression. An increasing use of traditional Chinese medicine for depression treatment showed that traditional prescription drugs exhibited certain clinical efficacy, enhanced efficacy, and reduced dosages and side effects of common medicines, in combination with other antidepressants [8]. In Chinese medicine theory, liver plays an important role in the pathology of depression. Depression is commonly caused by emotional stress and injury as well as failure of liver catharsis function and stagnation of liver Qi, (Qi is an energy flow, a vital energy, that circulates the body to regulate body functions) or life source [9–11]. Shuyusan has been effective herbs prescription against depression in our hospital for many years, which shows the effect of purifying the heart heat and regulating Qi and Blood. Our previous clinical study showed that Shuyusan could improve the clinical symptoms of depression, and laboratory study indicated that Shuyusan could increase 5-hydroxytryptamine (5-HT) and improve the 5-HT expression of hippocampus neurons on rats caused by chronic mild unpredictable stress-induced depression [12–14]. The main component of Shuyusan was Geniposide, Deoxyschizandrin, and Spinosae flavonoid glycosides. The Geniposide has a protective effect for SH-SY5Y cells, which injured by the high-dose corticosterone injury model using SH-SY5Y cells [15]. Therefore, protecting neurotransmitters from injury is one of the most important neuroprotective roles of Shuyusan whether the antidepressant activity effect of Shuyusan is dependent on its interaction with GR receptors in the hippocampus and could regulate the serum level of CRH, ACTH, and CORT in the rat of chronic stress-induced depression. In the present study, we aimed to investigate the effect of Shuyusan on the behavioral despair tasks, serum level of CRH, ACTH, and CORT, and expression of glucocorticoid receptor of HPA in current study.

## 2. Materials and Methods

### 2.1. Animals

We used 70 adult male Sprague-Dawley (SD) rats weighed 180–220 g (license no. SLXK 2009-0007) for quantitative analysis. SD rats were supplied by the medical experimental animal center of the Chinese People Liberation Army (PLA) General Hospital. The 70 SD rats were equally and randomly assigned into one of six groups, namely, normal control, model, high-dose treatments, medium-dose treatments, low-dose treatments with Shuyusan decoction, and fluoxetine treatment group (*N* = 10). In addition, traditional Chinese medicine and Western medicine treatment groups were administered by Shuyusan herbs decoction and fluoxetine, respectively. Model and control groups were treated with saline. One rat in the traditional Chinese medicine treatment group died at 24 days and hence was excluded from analysis, but all remaining rats were included in the final analysis. Protocols were conducted in accordance with the Guidance Suggestions for the Care and Use of Laboratory Animals, formulated by the Ministry of Science and Technology of the People's Republic of China.

### 2.2. Drugs, Reagents, and Drug Administration

Shuyusan (10 g *Bupleurum*, 15 g Radix Curcumae, 6 g mint, 10 g cape jasmine fruit, 10 g *Poria cocos*, 10 g radix polygalae, 10 g calamus, 15 g spine date seed, and 10 g flower of silk tree *Albizzia* in Table 1) was provided by the pharmacy of the PLA General Hospital. Its main active compounds are spine date seed, magnoliavine fruit, and cape jasmine fruit. Shuyusan was boiled, filtrated and concentrated to 5 g/mL liquided at 80°C water bath, and stored at 4°C refrigerator. When used, it was diluted with distilled water gavages to rats. Prozac (fluoxetine hydrochloride dispersible tablets, pantheons France company, France, Lot: 9711B, 20 mg/tablet), was used in concentration to 2 mg/mL liquid by distilled water. The control group was normally fed without any treatment. The rats in the model, Shuyusan groups, and fluoxetine group were subjected to a model of unpredictable chronic mild stress and fed isolation. The model group was fed normally after 21-day stress stimulation; high-dose Shuyusan group was administered with Shuyusan contained the herds of 25 g/kg by gastric perfusion one time per day; medium-dose Shuyusan group was with the herds of 7.5 g/kg; low-dose Shuyusan group was with herds of 2.5 g/kg; fluoxetine was dissolved into 2 mL normal saline and was administered 10 mg/kg through gastric perfusion for the fluoxetine group. The fed dosage of Shuyusan: according to clinical experience, the effective dosage of normal 60 kilogram adult was to take 96 gram per day of Shuyusan, so the rat of 200 gram would take Shuyusan 0.32 gram per day. The rats started accepting the above treatments in the 21 days and the rats in treatment groups were continuously administered with the drugs through gastric perfusion for 21 days. All rats were included in the final analysis. Rat CRH, ACTH, and CORT ELISA kit (R&D, United States), GR primary antibody kit (Santa Cruz), low temperature for high-speed centrifuge (Sigma, USA), inverted microscope (OLYMPUS Japan), microplate reader (TECAN, Switzerland), homemade open boxes, and so forth.

### 2.3. Model of Depression for Chronic Unpredictable Mild Stress (CUMS)

Adopted model of depression for chronic unpredictable mild stress (CUMS) and improvement for a little. The normal control group was housed five rats per cage and dieted, drunk water, not to any stimulus. The model group was fed one rat for each cage and received 21 days of stress stimulation, that contains water deprivation (24 h), fasting (24 h), clamping the tail (1 min), electric shock foot (20 v, 10 seconds/time, 1 min), forcing swim in ice water (4°C, 4 min), tilting cages (45°C, 12 h), converting the circadian rhythm, braking (2 h), and thermal stimulation (40°C, 5 min), randomly assigned 1-2 kinds of stimulation daily, and each stimulus repeated 2-3 times.

### 2.4. ELISA Analysis of CRH, ACTH, and CORT

After the end of the experimental period, all rats were intraperitoneally injected for anesthesia by 10% chloral hydrate (3 mL/kg), exposed heart, perfused rinse through the ascending aorta by 0.9% saline (37°C, 250 mL) and perfuse 4% paraformaldehyde solution (4°C, PH = 7.4, 200 mL) until limbs stiff of the animals. Finally, the brains were removed and repaired block, stored in 10% polyformaldehyde solution. The organization of the brain tissue was added an appropriate amount of saline and mashed, centrifuged for 10 min (3000 rpm), and extracted the supernatant. Then we removed the ELISA kit (CRH, ACTH, and CORT) from 4°C refrigerator and coordinated to room temperature, removed the reaction plate, and set blank wells separately (blank comparison wells did not add sample and HRP-conjugate reagent, other each step operation is same). The sample test well: we added sample dilution 40 *μ*L to testing sample well, then added testing sample 10 *μ*L (sample final dilution is 5-fold), added sample to wells, did not touch the well wall as far as possible, and gently mix. After closed plate with closure plate membrane, we incubate for 30 min at 37°C, then uncovered closure plate membrane, discarded liquid, dried by swing, add washed buffer to every well, still for 30 s then drained, repeated 5 times, and dried by pat. Then added HRP-conjugate reagent 50 *μ*L to each well, except blank well; added chromogen solution A 50 *μ*L and chromogen solution B to each well, evaded the light preservation for 15 min at 37°C; added stop solution 50 *μ*L to each well; stop the reaction. We took blank well as zero, read absorbance at 450 nm after added stop solution and within 15 min. In the end, we detected the concentration in the standard curve according to OD values of samples.

### 2.5. Behavior Despair Study

Open-field test: an open-field method was used to conduct praxeological scoring in all group rats. The open-field device was made of opaque materials with 76 cm × 76 cm square, located on the bottom, which was equally divided into 25 equilateral squares. Around, there was a wall with the height of 45 cm. The rat was put in the central square and then measured the square numbers the rat crossed in 3 minutes (only the squares the rat entered on four feet could be numbered as the score of horizontal activity) and the times of standing on hind limbs were observed. Each rat was measured once for three minutes, which would be scored by two observers and the average value was taken. The percentage time spent in this central zone is considered indicative of exploratory behavior and may reflect a decrease in anxiety, although this OF parameter is not sensitive to all anxiolytics and may not model certain features of anxiety disorders [16, 17].

**Forced-Swimming Test (FST)**

A forced-swimming test was used to all groups, according to the method of Porsolt [18]. Rats were placed in an open cylindrical container (diameter 10 cm, height 25 cm), containing 15 cm of water at 25 ± 1°C. The duration of observed immobility was recorded during the last 4 min of the 6 min testing period [19, 20]. Rats are forced to swim in the restricted space from which they cannot escape and are induced to the characteristic behavior of immobility. The duration of observed immobility was recorded during the last 4 min of the 6 min testing period. When rats ceased struggling and remained floating motionless in the water, they were judged to be immobile. Decrease in the duration of immobility during the FST was taken as a measure of antidepressant activity.

**Tail Suspension Test (TST)**

The duration of immobility time induced by tail suspension was measured according to the method of Steru [21]. Rats both acoustically and visually isolated were suspended above the floor by adhesive tape placed approximately 1 cm from the tip of the tail. The remained immobile time of TST was quantified for 6 min. Rats were considered immobile only when they hung passively and completely motionless.

### 2.6. GR-Receptor Determination

Endogenous peroxidase was inactivated with 3% H_2_O_2_. Sections were blocked in 10% normal goat serum at 37°C for 30 minutes, added primary antibody at 4°C overnight, and washed with PBS. The average optical density of positively stained cells of the slices obtained above was analyzed using Image-Image Pro software (Media Cybernetic, Bethesda, Maryland, USA) to analyse the immunohistochemical positive cells and integrated optical density (integral optical density, IOD). Three slices from each group were chosen for the analysis. For each slice, three images from three different areas, the layer four to layer five of frontal cortex, region CA1 of hippocampus (located about 600 um away from the starting point of middle line of A1 area) and region CA3 of hippocampus (the top point of hippocampus turning area), were evaluated under 200x objective lens.

### 2.7. Statistical Analysis

All results are calculated with means ± SEM. The variance analysis was used to compare among groups. If *P* value is less than 0.05, the difference was considered statistically significant. We use test for homogeneity to examine the data: if the data is homogeneous, we conduct analysis of variance (one-way ANOVA) directly on the data. Between the two groups, we used the LSD method to compare any difference. Otherwise, we changed parameters first and then used test for homogeneity again. We only conduct ANOVA on data which becomes homogeneous after change of parameters. All statistical analyses were carried out by using SPSS for Windows (SPSS Inc.).

## 3. Results

### 3.1. The Open-Field Test

**Figure 1:**
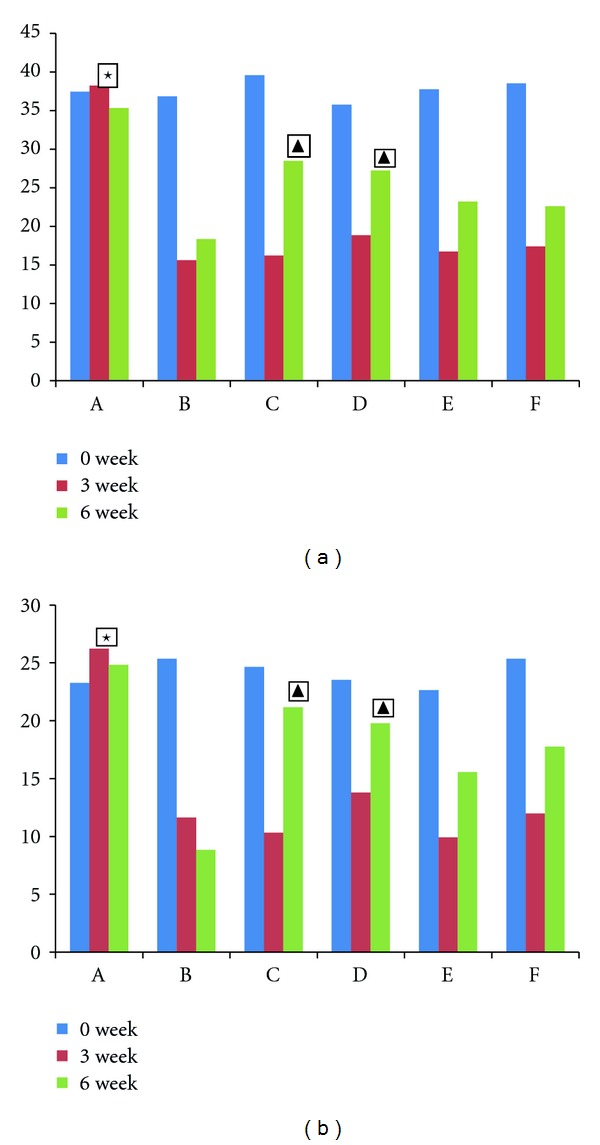
Observation of rats on open-field activities (horizontal, vertical). Horizontal movement scores reflect range of motion; vertical movement scores reflect exploratory behaviors. High scores represent high degree of activity. A: normal group, B: model group, C: fluoxetine group, D: high-dose Shuyu group, E: medium-dose Shuyu group, and F: low-dose Shuyu group. All data are expressed as the $\bar{x}\pm s$, (*n* = 10). ★*P* < 0.05 versus other groups; ▲*P* < 0.05 versus model group.

The results of the open-field test are reported in Figure 1. It shows observation on activity of rats (horizontal, vertical). There was no significant difference before modeling in among groups (*F* = 1.307, *P* > 0.05). After modeling, it was observed that there was significant changes in the open-field test in these groups. After 3 weeks herbs fed treatment with high-dose herbs and fluoxetine, the immobility time was increased significantly, compared with the model group (high-dose group versus the model group *t* = 2.273, *P* < 0.05; fluoxetine group versus the model group *t* = 3.461, *P* < 0.01).

### 3.2. The FST in Each Group

**Figure 2:**
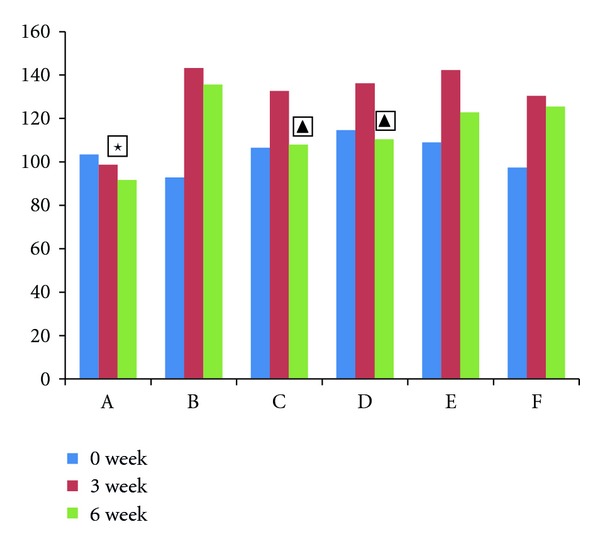
The FST in each group. Effects of immobility time during the forced swimming test. The vertical axis shows the immobility time (sec). High scores represent high degree of depression. A: normal group, B: model group, C: fluoxetine group, D: high-dose Shuyusan group, and E: medium-dose Shuyusan group. F: low-dose Shuyusan group. All data are expressed as the $\bar{x}\pm s$, (*n* = 10). ★*P* < 0.05 versus other groups; ▲*P* < 0.05 versus model group.

The results of FST are reported in Figure 2. There was no significant difference before modeling in among groups (*F* = 1.77, *P* > 0.05). After modeling, it was observed that there was a significant prolongation of immobility time in these groups. After 3 weeks herbs fed treatment with high-dose herbs and fluoxetine, the immobility time was increased significantly, compared with the model group (high-dose group versus the model group *t* = 2.575, *P* < 0.05; fluoxetine group versus the model group *t* = 3.061, *P* < 0.01).

### 3.3. The TST in Each Group

**Figure 3:**
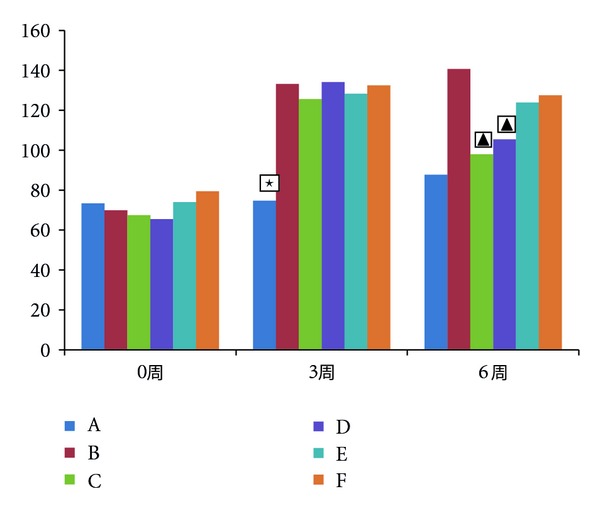
The TST in each group. Effects of immobility time during the tail suspension test. The vertical axis shows the immobility time (sec). High scores represent high degree of depression. A means normal group, B means model group, C means fluoxetine group, D means high-dose Shuyusan group, E means medium-dose Shuyusan group, and F means low-dose Shuyusan group. All data are expressed as the $\bar{x}\pm s$, (*n* = 10). ★*P* < 0.05 versus other groups; ▲*P* < 0.05 versus model group.

The results of pooling all the tests performed with TST are reported in Figure 3. There was no significant difference before modeling among groups (*F* = 1.05, *P* > 0.05). After modeling, it was observed that there was a significant prolongation of immobility time in these groups. High-dose Shuyusan herbs also could reduce the immobility time in TST. After high-dose herbs and fluoxetine treatment for 3 weeks, the immobility time was increased significantly, compared with the model group (high-dose group versus the model group *t* = 2.763, *P* < 0.05; fluoxetine group versus the model group *t* = 3.182, *P* < 0.01).

### 3.4. Determination of Serum Contents Level of CRH, ACTH, and CORT

**Figure 4:**
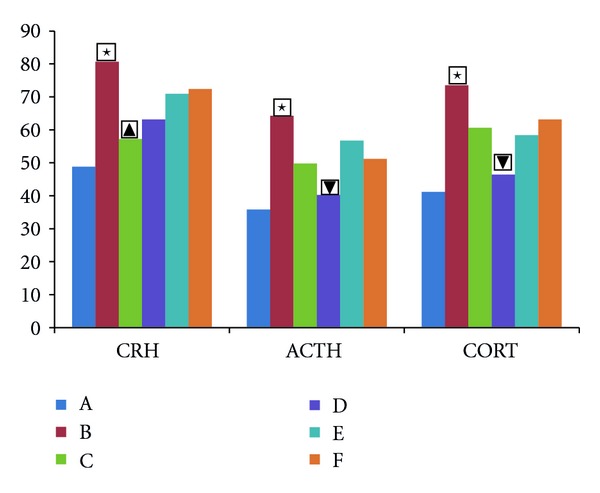
The serum contents level of CRH, ACTH, and CORT. The serum contents level of CRH, ACTH, and CORT in rats. The vertical axis shows the value. High scores represent high degree of depression. A: normal group, B: model group, C: fluoxetine group, D: high-dose Shuyu group, E: medium-dose Shuyu group, and F: low-dose Shuyu group. All data are expressed as the $\bar{x}\pm s$, (*n* = 10). ★*P* < 0.05 versus normal groups; *▼P* < 0.05 versus model group.

Figure 4 shows the serum contents level change of CRH, ACTH, and CORT in each group after treatment. The serum contents level in mode group was significantly increased for CRH, ACTH, and CORT (CRH: the model group versus the normal group *t* = 2.893, *P* < 0.01; ACTH: the model group versus the normal group *t* = 2.904, *P* < 0.01; CORT: the model group versus the normal group *t* = 3.646, *P* < 0.01). After 3 weeks herbs fed treatment, the serum contents level of CRH, ACTH, and CORT in high-dose group was significantly decreased, compared to the model group (CRH: high-dose group versus the model group *t* = 2.261, *P* < 0.05; ACTH: high-dose group versus the model group *t* = 2.387, *P* < 0.05; CORT: High-dose group versus the model group *t* = 2.478, *P* < 0.05). Although the serum contents level of CRH, ACTH, and CORT was decreased in the low-dose group and the fluoxetine group, there was no significant difference compared to the model group.

### 3.5. Expression of GR Receptor in the Rat Hippocampus

**Figure 5:**
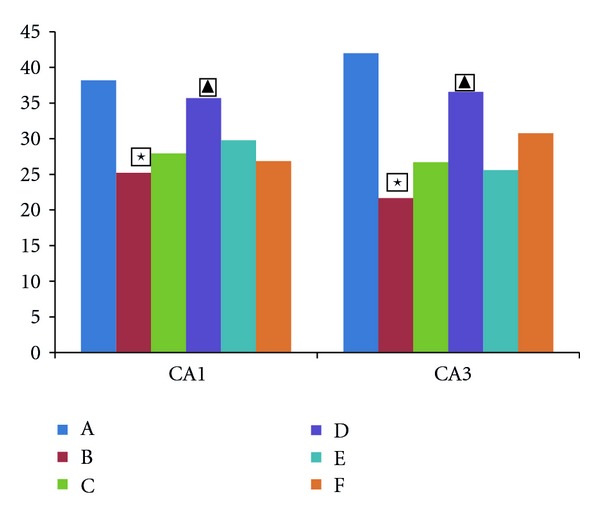
Expression of GR-receptor-positive cells in rat hippocampus. Comparison of GR-positive cells in rat hippocampus. The vertical axis shows the value (*n*). Low value represent high degree of depression. A: normal group, B: model group, C: fluoxetine group, D: high-dose Shuyu group, E: medium-dose Shuyu group, and F: low-dose Shuyu group. All data are expressed as the $\bar{x}\pm s$, (*n* = 10). ★*P* < 0.05 versus normal groups. ▲*P* < 0.05 versus model group. *▼P* < 0.01 versus model group.

**Figure 6:**
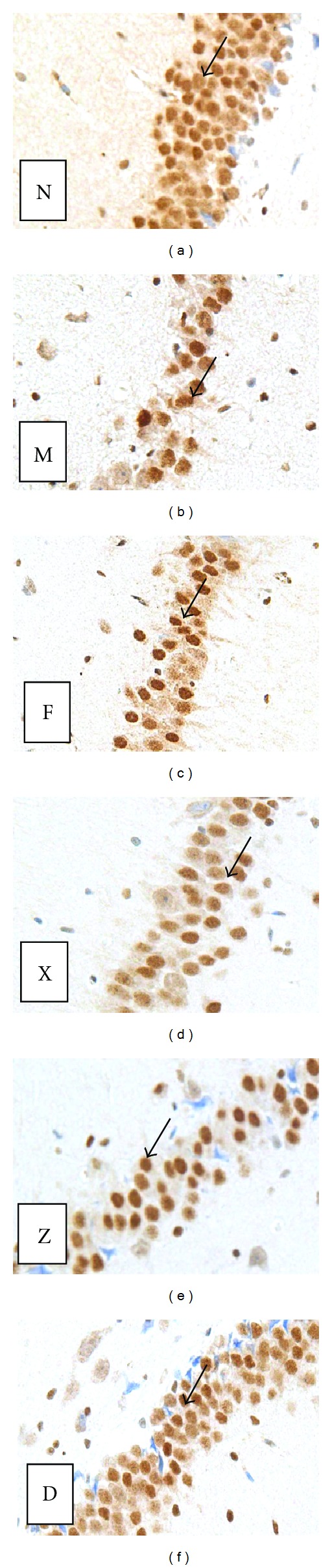
Expression of GR receptor in the rat hippocampus in the CA1 region. Changes in GR expression in the rat hippocampal CA1 region (immunohistochemistry, ×400). (N) GR expression was normal in the control group. (M) GR expression was subdued in the model group. (F) GR expression was enhanced in the fluoxetine group. Arrows indicate GR positive neurons. (D) GR expression was enhanced in the high-dose Shuyusan group. Arrows indicate GR positive neurons. (Z) GR expression was enhanced in the medium-dose Shuyusan group. Arrows indicate GR positive neurons. (X) GR expression was enhanced in the low-dose Shuyusan group. Arrows indicate GR positive neurons.

**Figure 7:**
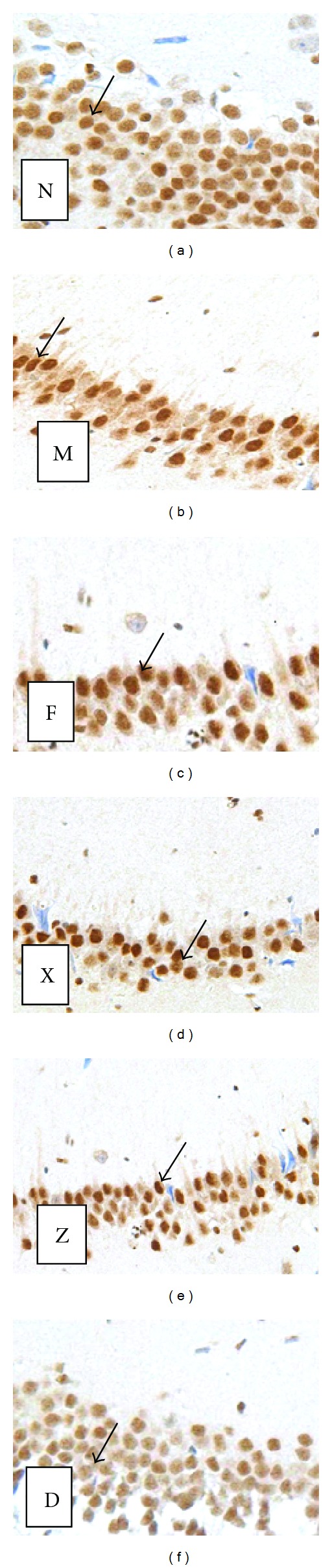
Expression of GR receptor in the rat hippocampus in the CA3 region. Morphology Changes in GR expression positive cells in the hippocampus of chronically stressed rat following treatment with Shuyusan in the rat hippocampal CA3 region (immunohistochemistry, ×400). The area of GR-positive cells in the hippocampus was decreased in the model group, and most of the cells were shrunk or lightly stained. The area of GR-positive cells in the hippocampus was increased in the M- and H-Shuyusan groups. (N) GR expression was normal in the control group. (M) GR expression was subdued in the model group. (F) GR expression was enhanced in the fluoxetine group. Arrows indicate GR positive neurons. (D) GR expression was enhanced in the high-dose Shuyusan group. Arrows indicate GR positive neurons. (Z) GR expression was enhanced in the medium-dose Shuyusan group. Arrows indicate GR positive neurons. (X) GR expression was enhanced in the low-dose Shuyusan group. Arrows indicate GR positive neurons.

Figures 5, 6, and 7 show the number of GR-receptor-positive cells in the hippocampus of rats in each group. The GR-receptor positive cells in the hippocampus of rats in each group change of GR for chronic unpredictable mild stress mainly manifested in the areas of CA1 and CA3 of the hippocampus (CA3 region is more prominent). GR-positive-cells in the hippocampus of normal group arranged in dense, structured, and distributed in normal. The immune response in hippocampus of model rats was significantly decreased, GR-positive cells arranged loose, and less structured. The number of GR-receptor-positive cells was significantly decreased compared to the mode group (CA1 region: the model group versus the normal group *t* = 2.713, *P* < 0.05; CA3 region: the model group versus the normal group *t* = 5.275, *P* < 0.01). The number of GR-receptor-positive cells was significantly increased compared to the model group in the CA1 region and CA3 region (CA1 region: high-dose group versus the model group *t* = 2.157, *P* < 0.05; CA3 region: high-dose group versus the model group *t* = 3.257, *P* < 0.01). The medium-dose group, low-dose group, and the fluoxetine group all have increased trend for the contents of the number of positive cells and IOD, but there was no significant difference.

## 4. Discussion 

Behavioral studies play an important role in the evaluation of antidepressant activity effect [22]. The forced swimming and tail suspension tests are behavioral despair tests, and it is useful for probing the pathological mechanism of depression and for the evaluation of antidepressant drugs [23]. FST and TST had been widely used as preclinical screening tool of antidepressant drugs [24, 25]. Characteristic of rat behaviors scored in both tests is termed immobility, which reflects behavioral despair as seen in human depression [26]. The duration of immobility time in rats that are trapped and forced to swim is closely related to helplessness. It was observed from our results that there was a significant prolongation of immobility time in these groups after modeling; with high-dose herbs of Shuyusan administer, the immobility time increased significantly. It suggests that Shuyusan has effect in producing significant antidepressant-like activity, when assessed in FST and TST. Based on the traditional Chinese medicine theories, as well as achievements from modern scientific studies and clinical trials, Shuyusan exhibits therapeutic effects on depression by purifying the heart heat, resolving phlegm, and regulating Qi and blood. Previous animal studies have revealed the antidepressant effects of Shuyusan based on behavioral improvement in rat models of depression [11, 13].

Many studies suggested that depression is related to psychological, neuron-endocrine disorders, imbalances of monoamine neurotransmitter, dysfunction of hippocampus neurogenesis, and many other factors [27–30]. Long-time stress and elevated glucocorticoid levels leaded to the emergence of mood disorders [31]. Steroid hormones can modulate neuronal transmission by a variety of mechanisms. Hippocampus is rich in glucocorticoid receptors, the neurons of this area can be damaged by the high level of glucocorticoid; in the pathogenesis of depression studies, HPA axis response stress becomes the focus [32, 33]. Beside monoamine neurotransmitters, the serum level of CRH, CORT, and ACTH and changes in levels of GR were being used to evaluate depression [24–26]. Stress is under the influence of external environmental stimuli and could awaken the internal psychological corresponding; this response occurs through the nervous system and hormonal system [34, 35]. HPA hyperactivity is a major pathology-physiological factor for the depression, the continuous increased CORT in combined with GR overexpressions which in the hippocampus damage the hippocampus and locus coeruleus, so leads to atrophy and apoptosis of hippocampus neurons [36, 37]. In addition, hyperactivity of the HPA axis during stress is inhibited by GR, so that it returned to baseline levels. The effect of the GR reduced after it damaged so that HPA axis is more hyperactive and form a vicious cycle [38]. On the other hand, as hyperactivity of HPA axis, the 5-HT content is inhibited. As high concentration of CORT could induce the liver to produce tryptophan pyrrole enzyme, it could degradate the tryptophan in the blood. Tryptophan is the precursor of 5-TH, its reduction could lead to synthesis of 5-TH, so led to 5-HT content decrease in the brain, thus causing depressive symptoms [39, 40]. In physiological conditions, the secretion of CRH and ACTH regulated by the 5-TH system. In a stress state, HPA axis function becomes hyperactive and 5-HT synthesis significantly decreases as a result of insufficient tryptophan transported into the central nervous system [41–43]. Results from the present study demonstrated that Shuyusan could decrease the serum contents of CRH, ACTH, and CORT. It also could increase the expression of hippocampus GR receptor in the rat model of depression. Our results suggested that the mechanisms of action of Shuyusan were due to decreasing the serum contents level of CRH, ACTH, and CORT and increasing the expression of hippocampus GR receptor. Fluoxetine is selective serotonin reuptake inhibitors' (SSRIs) medications, it could increase the levels of NE, 5-HT, and DA in the brain of rat and was related to downregulation of 5-HT receptor, but not decreased the serum contents level of CRH, ACTH, and CORT and increased the expression of hippocampus GR receptor.

The chronic stress-induced depression model is an effective model for studying depression and has been widely utilized in basic research and drug screening for depression [44, 45]. The model can simulate the core symptoms of depression, that is, loss of interest, anhedonia, and decrease of exploring ability and sexual behavior. In fact, helplessness and anhedonia is the core symptom of depression and most of the current models only mimic anhedonia. The currently available chronic mild stress model is probably the most valid and the most widely used animal model of depression. Presently, the FST is the most widely used tool in depression research [45]. The experiment shows that the immobility time in forced swimming extends. That proved the replication model of depression in rats were succeed.

In conclusion, our data indicated that high-dose herbs Shuyusan had antidepressant activity effect on chronic stress-induced depression model rats. The behavioral indicators improved compared with model group. We found that CRH, ACTH, and level of CORT in serum and GR expression in hippocampus of the high-dose herbs Shuyusan group were significantly improved, compared with the model group by ELISA and immunohistochemical methods. Thus, we confirmed that Shuyusan has antidepressant activity effect, its mechanism may be related to decreasing glucocorticoid levels activity, regulating the function of HPA axis, and inhibiting glucocorticoid receptor expression in hippocampus.

## Figures and Tables

**Table 1:** The table of components and ratio of Shuyusan.

Plant species	Family	Plant part	Pinyin
*Bupleurum*	Umbelliferae	Root	Chaihu
*Radix curcumae*	Zingiberaceae	Root	Yujin
Mint	Labiatae	Leaf	Bohe
Cape jasmine fruit	Rubiaceae	Fruit	Zhizi
*Poria cocos*	Polyporaceae	Sclerotium	Fuling
Radix polygalae	Polygalaceae	Root	Yuanzhi
Calamus	Araceae	Rootstock	Shichangpu
Spine date seed	Rhamnaceae	Seed	Suanzaoren
Flower of silk tree *Albizzia *	Pea family	Inflorescence	Hehuanhua
