# Correction to “Antidepressant‐Like Effects of Shuyusan in Rats Exposed to Chronic Stress: Effects on Hypothalamic‐Pituitary‐Adrenal Function”

**DOI:** 10.1155/ecam/9896170

**Published:** 2026-08-03

L. Chen, M. Chen, F. Wang, Z. Sun, H. Quanzhi, M. Geng, H. Chen, and D. Duan, “Antidepressant‐Like Effects of Shuyusan in Rats Exposed to Chronic Stress: Effects on Hypothalamic‐Pituitary‐Adrenal Function,” *Evidence-Based Complementary and Alternative Medicine*, vol. 2012 (2012), https://doi.org/10.1155/2012/940846.

In the article titled “Antidepressant‐Like Effects of Shuyusan in Rats Exposed to Chronic Stress: Effects on Hypothalamic‐Pituitary‐Adrenal Function,” there was an error in Figure 7.

**FIGURE 7:**
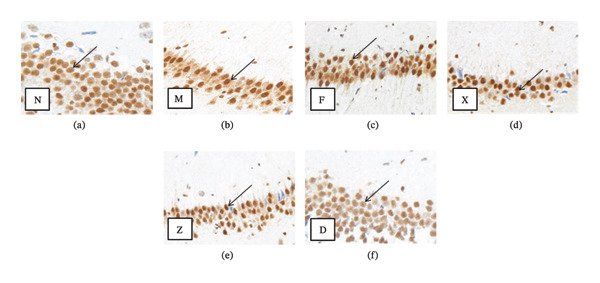
Expression of GR receptor in the rat hippocampus in the CA3 region. Morphology changes in GR expression positive cells in the hippocampus of chronically stressed rat following treatment with shuyusan in the rat hippocampal CA3 region (immunohistochemistry, × 400). The area of GR‐positive cells in the hippocampus was decreased in the model group, and most of the cells were shrunk or lightly stained. The area of GR‐positive cells in the hippocampus was increased in the M‐ and H‐shuyusan groups. (N) GR expression was normal in the control group. (M) GR expression was subdued in the model group. (F) GR expression was enhanced in the fluoxetine group. Arrows indicate GR positive neurons. (D) GR expression was enhanced in the high‐dose shuyusan group. Arrows indicate GR positive neurons. (Z) GR expression was enhanced in the medium‐dose shuyusan group. Arrows indicate GR positive neurons. (X) GR expression was enhanced in the low‐dose shuyusan group. Arrows indicate GR positive neurons.

More specifically, the image of the GR expression in the neurons from the fluoxetine group in panel (c) was incorrect. This error was introduced by the authors during manuscript preparation and Figure 7 should be corrected as follows:

We apologize for this error.

